# Supplementary material for: Multi-packet transmission aero-engine DCS neural network sliding mode control based on multi-kernel LS-SVM packet dropout online compensation
Source: PLoS One. 2020 Jun 17;15(6):e0234356. doi: 10.1371/journal.pone.0234356 (PMC7299384; doi:10.1371/journal.pone.0234356)
Supplement: Attachment — Submitted filename: Modification Discription.docx [file pone.0234356.s002.docx]

Modification Discription

Dear Editor:

In view of the relevant issues raised by you, the specific modifications are as follows:

Q1:*Please explain the rationale for the development of your system in light of recent research in this area, clearly indicating which problem with existing system you are addressing.

R1:In recent years, aero-engine distributed control system has been widely concerned by scholars at home and abroad. Compared with the centralized control, the signal transmission in the distributed control system is completed by the bus network. The low-level processing functions in the central processor, such as signal acquisition, excitation, demodulation / decoding, filtering, etc., are placed in the intelligent sensor, while the intelligent actuator completes the closed-loop feedback, operation mode control, self-test, redundancy management and other functions; the central controller only Complete advanced functions, such as control plan, control algorithm, network scheduling, status monitoring, etc. By using distributed sensor, actuator and bus network, the system weight is greatly reduced, the control performance and reliability are greatly improved, the development cost is significantly reduced, and the development cycle is greatly shortened.

At present, the research of distributed control system mainly includes node driving mode, network induced delay, packet loss, communication restriction, multi packet transmission and so on.

Relevant information has been added into the introduction.

Q2:*Please clearly report at the beginning of your methods or results section which were the key performance measures used to establish the validity and utility of your system. Please also report clearly which statistical analysis was used to establish robustness of performance measures.

R2:In this method, the controller is designed for the multi packet transmission network control system. Therefore, the effect of packet loss compensation is mainly determined by the error of the actual data and the data under the condition of relevant packet loss rate. The smaller the error is, the better the compensation effect is.

For the sliding mode controllers with different reaching laws, the response time and steady-state error are mainly used to determine the quality of the controller. The shorter the response time, the better the control speed and the smaller the steady-state error, the better the chattering suppression effect and the higher the precision of the sliding mode controller.

Relevant performance index descriptions have been added to the results section

Q3:*Whilst we note that the performance of your system has been compared to other methods, the manuscript is lacking a thorough discussion of the different laws chosen for comparison and justification as to why they were chosen. Please modify your manuscript as necessary.

R3:In this paper, the comparative law of approach, which includes the fixed parameter PID law of approach, the fuzzy law of approach, the piecewise law of approach, the exponential law of approach, and the global law of approach are choosed. These laws are all traditional laws of approach. Because of their exponential characteristics, the exponential law of approach is famous for its fast response speed. The piecewise law of approach is achieved by considering the system performance in different time periods and applying different characteristics of the law of approach In a certain performance, the fuzzy exponential approach law realizes superior sliding mode control by adjusting relevant parameters on-line by fuzzy theory, and the global approach law realizes control by considering global characteristics. All of the above approaches have been proved to be effective and widely used. The fixed parameter PID approach law mainly compares the advantages of RBF-PID adaptive adjustment.

Relevant descriptions have been added to the results section.

In addition, relevant data of all simulation diagrams in the article has been sent to you in the form of appendix, please check. Since most of the data in this paper are generated by MATLAB, the format of related data is .mat.Among them, the data in Figure 10-15 has been completely arranged and placed in the excel table, and the corresponding other data are matlab table files.

Yours sincerely

Guangfu Li
